# Syntactic Errors in Older Adults with Depression

**DOI:** 10.3390/medicina59122133

**Published:** 2023-12-07

**Authors:** Chengjie Xu, Nahathai Wongpakaran, Tinakon Wongpakaran, Teeranoot Siriwittayakorn, Danny Wedding, Pairada Varnado

**Affiliations:** 1 Master of Science Program in Mental Health, Multidisciplinary and Interdisciplinary School, Chiang Mai University, Chiang Mai 50200, Thailand; chengjie_xu@cmu.ac.th (C.X.); tinakon.w@cmu.ac.th (T.W.); teeranoot.s@cmu.ac.th (T.S.); danny.wedding@gmail.com (D.W.); 2 Department of Psychiatry, Faculty of Medicine, Chiang Mai University, 110 Intawaroros Rd., T. Sriphum, A. Muang, Chiang Mai 50200, Thailand; pairada.v@cmu.ac.th; 3 Department of English, Faculty of Humanities, Chiang Mai University, 239, Huay Kaew Road, Muang District, Chiang Mai 50200, Thailand; 4 School of Humanistics and Clinical Psychology, Saybrook University, Oakland, CA 91103, USA

**Keywords:** major depressive disorder, syntax, older population, late-life depression, linguistic, cognitive

## Abstract

*Background and Objectives*: This study investigated the differences in syntactic errors in older individuals with and without major depressive disorder and cognitive function disparities between groups. We also explored the correlation between syntax scores and depression severity. *Materials and Methods*: Forty-four participants, assessed for dementia with the Mini-Cog, completed the 15-item Geriatric Depression Scale (TGDS-15) and specific language tests. Following a single-anonymized procedure, clinical psychologists rated the tests and syntax scores. *Results*: The results showed that the depressive disorders group had lower syntax scores than the non-depressed group, primarily on specific subtests. Additionally, cognitive test scores were generally lower among the depressed group. A significant relationship between depression severity and syntax scores was observed (r = −0.426, 95% CI = −0.639, −0.143). *Conclusions*: In conclusion, major depressive disorder is associated with reduced syntactic abilities, particularly in specific tests. However, the relatively modest sample size limited the sensitivity of this association. This study also considered the potential influence of cultural factors. Unique linguistic characteristics in the study’s context were also addressed and considered as potential contributors to the observed findings.

## 1. Introduction

The global demographic landscape is undergoing significant changes. The number of elderly people is experiencing a substantial and drastic increase [1]. Among the complexities that older individuals encounter, late-life depression is one of the concerning healthcare problems [2] that significantly impacts quality of life and increases the risk of suicide [3,4,5]. Various factors associated with depression differ or share similarities between young and older adults [6,7,8]. Depression is known to affect the means in which individuals feel, think, and communicate through language [9,10]. Language processing includes working memory and phonological, semantic, and syntactic processing [11]. Language is a complex and multifaceted tool for communication, encompassing various aspects such as morphology (the structure of words and how they are formed from smaller units), semantics (word meanings and their use in conveying meaning within sentences and discourse), syntax (the arrangement of words and phrases to create well-formed sentences), pragmatic (the use of language in context), and phonology (the sounds and pronunciation of words in a language). In individuals with depression, these components of language can undergo significant changes, reflecting the profound effects of the condition on their ability to express themselves and interact effectively with others.

Among language processing related to depression, semantics gains much attention from clinicians and researchers. Common verbal expression indicatives of semantic problems include a negative view of oneself and others [12,13].

Although syntax issues in individuals with depression have been extensively studied in English-speaking populations, there is a notable absence of research regarding syntactic problems in Thai-speaking older individuals with depression.

Studies have demonstrated that syntax used among individuals with depression differs from non-depressed individuals. For example, amongst nouns, verbs, and first-person singular pronouns, negative words were more frequently used than non-depressed individuals [14,15]. Research also found that depressed individuals tended to use past tense extensively for verb actions, inverted word order for topics, use of emphasis, presence of short, impersonal, truncated and arid sentences, and presence of ellipsis [16].

It is to be noted that Thai and English have different syntax and grammatical structures. For example, Thai follows a subject–verb–object (SVO) word order, similar to English. However, Thai is a pro-drop language, which means that pronouns are often omitted if they can be inferred from the context. Thai verbs are not conjugated for tense. That is, verb forms generally remain the same regardless of when an action occurrs. Instead, time expressions are used to indicate tense. Thai does not have articles (a, an, the) or gender-specific pronouns (he, she). The same pronoun, “เขา” (khau) is used for “he”, and “she” in Thai. Adjectives in Thai typically follow the noun they modify. They do not change based on gender, number, or case. Thai does not typically mark plurals with the addition of an “s” as in English. Plurality is often inferred from context. Thai is a tonal language with five tones. The tone of an otherwise identical string of sounds can changes its meaning [17].

A study by Angkapanich and Intasian demonstrated that depressed patients used a monologue style for writing daily diaries, like talking to themselves. They used the form of an abstract question sentence, which were questions that cannot be answered, such as “Why is it like this?”, the passive voice form, first-person pronouns, words expressing negative emotions, and negative words, and metaphors to compare themselves and others [18].

Given the fact that older adults are compromised to cognitive decline, depression may add up to language expression. A substantial body of research has established clear connections between language and depression. However, it is important to note that most of these studies have been conducted in younger individuals. Consequently, there exists a notable gap in the understanding of language alternations in older individuals with LLD. It is evident that the primary concerns in the study of language and its related issues often revolve around semantics, which pertains to the meanings conveyed by words and phrases, and pragmatics, which deals with how language is used effectively within various contexts. For example, participants with depression often used words like “hopeless”, “worthless”, and “helpless” to describe themselves [19]. They also use intensified adjectives such as “completely” to amplify their self-perceptions of negativity [20].

Syntax is one of the most complex cognitive tasks that humans routinely engage in: languages contain countless syntactic rules, which must be accessed rapidly and simultaneously during normal language processing. When considering syntax, it is important to recognize its complexity as it can differ significantly across cultures and languages. Additionally, it is worth noting that research on syntax is relatively scarce, particularly in the context of older adults.

Previous studies primarily focused on depressed adults in languages like English [21] and Russian [22], yet there is a dearth of research investigating this issue among older individuals. Another research gap is the absence of evidence demonstrating syntax-related issues among the older population with depression in Thai, which has distinct syntactic rules compared to languages examined in prior studies.

Unlike some studies of syntax using text analysis of the transcripts from interviews, notes, and diaries, the authors of this study employed psychological tests commonly used in geriatric clinics to examine syntax in older patients with depression. By analyzing the response from the tests, we had two objectives, one was uncovering potential disparities of syntax use, and the second was to identify which test was likely to be sensitive to the discrepancy between older adults with and without LLD. In addition, we also assessed other factors associated with depression, such as reaction time and time to respond to questions. The authors hypothesized that syntactic problems should be identified among depression group, as occurs in younger populations.

## 2. Materials and Methods

### 2.1. Design

This study was designed as a case–control study, targeting an older population. This study was conducted on-site at the Geriatric Psychiatry Clinic, Maharaj Nakorn Chiang Mai Hospital, Faculty of Medicine, Chiang Mai University, with a psychologist administering the test. This study was approved by the Institutional Review Board (or Ethics Committee) of the Faculty of Medicine, Chiang Mai University. The participants were divided into two groups based on their sex and age: a case group consisting of depressed patients, and a control group consisting of non-depressed individuals.

The diagnosis used for major depressive disorder (MDD) was based on the DSM-5 [23], where participants frequently experience the following symptoms nearly every day: depressed mood, markedly diminished interest, significant weight loss or weight gain, insomnia or hypersomnia, psychomotor agitation or retardation, fatigue or loss of energy, feelings of worthlessness or inappropriate guilt, diminished ability to think or concentrate, and suicidal thoughts or behaviors. Additionally, other major psychiatric disorders must be excluded from consideration.

The diagnosis was conducted by the geriatric psychiatrist (NW) through a clinical interview. Furthermore, to confirm the presence of depressive symptoms in the case group, the Geriatric Depression Scale (GDS) was utilized. Participants were required to score above the designated cutoff criteria on the GDS. Lastly, all participants were confirmed to have an absence of dementia.

Inclusion and Exclusion criteria: This study included participants up to 60 years old who had no history of significant neurocognitive disorder or dementia of any cause, visual or hearing impairment that would affect communication with the researchers, history or ongoing symptoms of psychotic disorder, or history or ongoing symptoms of bipolar disorder. Furthermore, participants with abnormalities in blood pressure, heart rate, or respiratory rate attributable to any cause, as well as those with physical or psychological conditions requiring emergent medical attention (such as suicide risk, severe agitation, or delirium), were excluded from the study.

After that, participants who received a diagnosis of major depressive disorder, a score of 6 or higher on the Thai version of the Geriatric Depression Scale [24], and those who scored 3 points or higher on a Mini-Cog test were included in the case group, whereas participants aged up to 60 years who scored less than 6 on the Thai version of the Geriatric Depression Scale and 3 points or higher on a Mini-Cog test were included in the control group.

The sample size estimation was derived from a related study conducted by Smirnova et al. [22], revealing that 73% of participants with depression, in contrast to 17% of healthy controls, exhibited reduced sentences when measuring syntactic variables. Using a 1:1 group ratio, a type I error (alpha, significance) of 0.05 was set, while the type II error (beta, 1-Power) was established at 0.10 (90% Power). Based on this, the estimated total sample size for both groups was determined as 30 (15 participants per group). However, due to differences in outcome assessments between this study and the aforementioned research, a larger dataset was deemed more advantageous for achieving adequate statistical power. Consequently, the researchers ultimately collected data from 44 participants (22 participants in each group).

**Figure 1:**
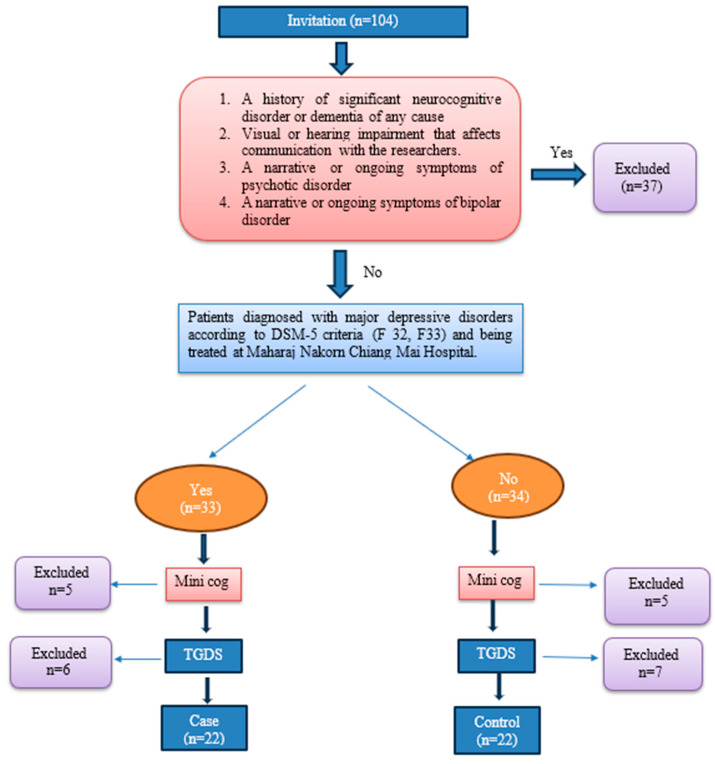
The flow chart of the study.

One hundred and four individuals were invited to participate in the study. Among all these people, 37 were excluded due to unmet criteria and unwillingness to participate, and then 23 were excluded because they did not meet the criteria for the GDS and Mini-Cog score, leaving 44 participants for the final analysis; 22 were in the case group and 22 in the control group (Figure 1). This study’s outcome was based on test scores, which comprised two parts: a syntax test and a cognition ability test.

### 2.2. Questionnaires and Measurements

#### 2.2.1. Questionnaires for Demographic Data

The data collected in this section included sex, age, marital status, income, dialect and alcohol use.

#### 2.2.2. Composited Neuropsychological Tests

The tests used as the composite tests in this study were derived from the standard intelligent test. All tests underwent confirmation of face validity by the research team, which included a geriatric psychiatrist (NW), a clinical psychologist (PV), and a linguistic expert (TS).

##### The Stanford–Binet Test (SB-5)

The Stanford–Binet test (SB-5) is a well-known cognitive function and intelligence test. The SB is an individual administration of intelligence and cognitive abilities. The Full-Scale IQ derives from administering ten subtests and is considered the standard measure of global intellectual ability. In this research, only one subtest was applied: Early Reasoning. The examinees were asked to describe and determine the cause and effect of events depicted in colorful illustrations. Three pictures were used: cat and ball, laundry, and puzzle. Each picture was scored 0, 1, or 2 points based on the test criteria. Response time was 30–40 s for each picture. The criterion validity for this instrument was confirmed, and it was administered by the investigator (PV), who held the test license.

##### Wechsler Adult Intelligence Scale—Fourth Edition (WAIS-IV)

Wechsler Adult Intelligence Scale—Fourth Edition (WAIS-IV) is a comprehensive clinical instrument for assessing the intelligence of examinees [25]. The WAIS-IV provides composite scores representing intellectual functioning in specified cognitive areas (i.e., Verbal Comprehension Index, Perceptual Reasoning Index, Working Memory Index, and Processing Speed Index) and a composite score representing general intellectual ability (i.e., Full-Scale IQ). For this study, only 1 subdomain was applied, the Verbal Comprehension Index, which comprises Similarities, Vocabulary, and Comprehension. The criterion validity for this instrument was confirmed, and it was administered by the investigator (PV), who held the test license.

Similarities Subtest

There are 18 pairs of items in this subtest. The examinee was presented with two words that represent everyday objects or concepts and describe how they are similar. It also involves crystallized intelligence, abstract reasoning, auditory comprehension, memory, associative and categorical thinking, the distinction between nonessential and essential features, and verbal expression [26,27,28,29]. Cronbach’s alpha of the present sample was 0.79.

Vocabulary Subtest

The Vocabulary subtest has 30 items, including 3 new picture items and 27 verbal items. Vocabulary is a core Verbal Comprehension test. For picture items, the examinee names the object presented visually. For verbal items, the examinee defines words presented visually and orally. Vocabulary is designed to measure an examinee’s word knowledge and verbal concept formation. It also measures an examinee’s crystallized intelligence, fund of knowledge, learning ability, long-term memory, and degree of language development [26,27,28,29]. Cronbach’s alpha of the present sample was 0.82.

Comprehension subtest

There are 18 items that were used in this study. The examinee answered questions based on his or her understanding of general principles and social situations. It is designed to measure crystallized intelligence, knowledge of conventional standards of behavior, social judgment, long-term memory, and common-sense expression [26,27,28,29]. Cronbach’s alpha of the present sample was 0.71.

#### 2.2.3. Syntax Score

The tests mentioned above were separately evaluated for syntactic accuracy and errors by the licensed clinical psychologists with a single-blind method. The scoring for syntax was as follows.

Score 2: The sentence was grammatically correct, demonstrating a good understanding of syntax rules, including word order (subject–verb–object), verb agreement (person, number, gender, and tense or mood), and sentence structure (subject, verb, object, modifier (e.g., adverb and adjective), clauses). No errors were present.

Score 1: The sentence contained some grammatical errors but was still partially correct. The errors may affect comprehensibility and language proficiency.

Score 0: The examinee could not construct a sentence or provide an answer.

#### 2.2.4. Mini-Cog Test

The Mini-Cog is a screening test for cognitive impairment [30]. It is a brief tool that helps detect dementia and even mild cognitive impairment. It includes three-word registration, clock drawing, and three-word recall. Word recall totals 3 points, and clock drawing totals 2 points. The final scores combine word recall and clock drawing. Participants receiving fewer than 3 points are considered to possess cognitive impairment. The Thai version has been widely used and has demonstrated good psychometric properties [31]. Cronbach’s alpha of the present sample was 0.78.

#### 2.2.5. Geriatric Depression Scale-15

The 15-item Geriatric Depression Scale (GDS-15) [24] is a test of screening for depressive symptoms in older adults. GDS-15 demonstrates good reliability and validity. The ranges of total scores were from 0 to 15. A score of 5 or above indicates depressive symptoms. The Thai version had been widely used and demonstrates good psychometric properties [32]. The Thai version of the Geriatric Depression Scale (TGDS) demonstrated good internal consistency (Cronbach’s alpha = 0.82). Regarding the test’s validity, the TGDS exhibited an Area Under the Curve of 0.88 in the predictive model assessing depression. This value indicates a robust discriminatory ability in distinguishing between individuals with and without major depression. Cronbach’s alpha of the present sample was 0.82.

### 2.3. Statistical Analysis

The case and control groups were matched regarding sex and age. Descriptive analysis, including frequency, mean, and standard deviation, was carried out for demographic and clinical data such as sex, age, education, and scores of syntax tools. The scores were compared between two groups using a *t*-test or Chi-square, depending on whether the type of variable was categorical or continuous.

Pearson’s correlation analysis was carried out for a correlational study between syntax scores and the severity of depression measured by the TGDS.

To explore the magnitude of mean differences in the test on the syntax score between depressed and non-depressed groups, the effect size was calculated using Cohen’s d, where d = 0.2 is considered a “small” effect size, while 0.5 represents a “medium” effect size, and 0.8 a “large” effect size.

In addition, to handle the small sample size, the bootstrap method was applied. With a small sample, it can be challenging to obtain reliable estimates of uncertainty, but bootstrap resampling allows us to do so without the need for complex mathematical approximations. It allows us to make statistical inferences and estimate the uncertainty of the results and a straightforward way to estimate confidence intervals for the statistics. In addition, if the data deviate from normality, as is often the case with small samples, traditional parametric methods may be less reliable. Bootstrap does not assume any specific distribution, making it robust for a wide range of data types. A percentile bootstrap was chosen to construct a confidence interval, such as the 2.5th and 97.5th percentiles for a 95% confidence interval. When the interval does not include a hypothesized value, such as zero, it might conclude that the result is statistically significant. A *p*-value 0.05 was considered significant, and IBM SPSS, version 27 (IBM Corp, Armonk, NY, USA), was used for the analysis.

## 3. Results

This study includes 22 individuals in the case group and 22 participants in the control group, who were meticulously matched for sex and age. In both groups, there were more females (9) than males (13), although this gender difference was not statistically significant (*p*-value = 1.00). The mean age in the case group was 67.22 ± 6.32, while in the control group, it was 67.86 ± 5.21. Statistical analysis revealed no significant age difference between the two groups (*p*-value > 0.05).

In the control group, seven participants (88%) held educational backgrounds that included at least a bachelor’s degree, whereas one participant (12.5%) had only completed elementary school. However, there was no significant difference in educational attainment between the groups (*p*-value > 0.05). Conversely, in the case group, seven participants possessed only an elementary school education, with only four individuals having a high educational background.

Approximately 68.8% of participants in the case group reported to have an income ranging from 0 and 5000 baht, while 35.3% of the participants in the control group fell into the same income bracket. Despite the majority of participants having incomes exceeding 2000 baht, it is noteworthy that more non-depressed participants had higher earnings compared to those with depression. However, statistical analysis indicated a non-significant income disparity between the groups (*p*-value > 0.05).

In the case group, 61.1% of the participants spoke northern Thai dialect, while 47.1% used standard Thai language. There were no significant differences in the language spoken between the two groups (*p*-value > 0.05).

The majority of participants, especially in the case group, resided with their families, the arrangement largely depending on their marital status. A smaller proportion of participants, primarily in this case group, were divorced or in other relationships and lived alone. However, there was no notable variation in marital status between the groups (*p*-value > 0.05).

Participants in the control group were more likely to have consumed alcohol in the year prior to the study. Nonetheless, there was no statistically significant difference in alcohol consumption between the groups (*p*-value > 0.05) (Table 1).

Regarding the TGDS and Mini-Cog, the case group had an average score of 4.09 ± 3.66, whereas the control group scored 1.59 ± 1.00, yielding *t* (42) = 3.086, with an effect size of 4.07 by Cohen’s d, indicating a very large effect. In contrast, the analysis of Mini-Cog scores in relation to MDD yielded a *p*-value of 0.268. This result indicated that there was no difference in cognitive function between the two groups.

Table 2 shows the syntax scores between the depressed and non-depressed group by each subtest; the case group demonstrated an average syntax score from the similarity test of 1.57 ± 0.31, while the non-depressed group exhibited an average score of 1.60 ± 0.23. However, it was not statistically significant. The same was true for the vocabulary and the Comprehension subtests; the effect size for the detection of a difference was small. In the Early Reasoning subtest, on the other hand, the case group exhibited a lower score of the tests than that in control group, especially the second picture of ‘Laundry’, revealing an almost large effect size in differentiating between the two groups (d = 0.785).

In the correlation analysis between the syntax score and TGDS, the syntax score is negatively associated with the TGDS score in the Comprehension and Early Reasoning tests (Table 3).

In the analysis of reaction times, no significant difference was observed, except for the reaction time for the test of the puzzle picture. The depressed group exhibited a mean and standard deviation of 6.31 ± 3.3, while the non-depressed group demonstrated a mean and standard deviation of 4.31 ± 3.91. The test difference using the *t*-test was *t* = 2.128, indicating that the difference in reaction times between these two groups was statistically significant (*p*-value < 0.05), with a moderate effect size (Table 4).

Table 5 shows the difference in the cognitive ability scores between the two groups. The depressed group significantly scored lower in all the tests with large effect sizes, except for the Early Reasoning test as the values did not include zero.

## 4. Discussion

The objective of this study was to investigate potential syntactic differences between depressed and non-depressed older individuals. The results of our study tend to support our initial hypothesis that individuals with depression achieve lower scores in syntax compared to those without depression.

However, among the four tests administered, only one exhibited statistical variance, specifically the Early Reasoning test. This finding aligns with a study conducted by Emery and Breslau [33], where not all neurological tests, but three out of eleven measures assessing syntactic complexity, showed significant differences between depressed and normal elderly individuals. Although a significant difference in syntax ability associated with depression was evident, it remains challenging to completely disregard the potential involvement of cognitive impairment. This consideration holds true whether the observed changes are temporary due to depression or indicative of cognitive decline associated with aging.

As previously mentioned, studies exploring syntax differences in older individuals, especially those with depressive symptoms, are scarce. The current study likely stands as one of the initial investigations conducted in older individuals diagnosed with major depressive disorder. The summarized findings substantiate the observation that older adults with major depressive disorder exhibit diminished proficiency in syntax usage. It is crucial to note that the outcomes are contingent on the chosen methodology and the neuropsychological tests utilized. This study highlights the Early Reasoning test as the most sensitive in discerning differences between depression and non-depression. In contrast, other tests like Similarity, Vocabulary, and Comprehension may necessitate larger sample sizes to detect disparities. For instance, the Comprehension test may require a sample size of 100 to achieve 80% power [34].

Regarding related studies conducted in adult populations, albeit differing from our sample, they warrant acknowledgment. In the adult population itself, findings tend to be inconsistent. Studies in Russian found that individuals with depression tend to use single-clause sentences, exhibit increased sentence reduction, and employ atypical or inverse word order more frequently than healthy control groups [22]. Similar patterns were observed in Trifu and colleagues’ study, where the depressed group displayed short and truncated sentences compared to the control group [16].

This syntactic difference can be attributed to difficulties in working memory and sustained attention that are often associated with depression [16]. This is in contrast to the study conducted by Schneider and colleagues that found no differences in their syntactic tests, e.g., pure syntactic complexity, simple sentences, coordinated sentences, and syntactic diversity between healthy controls and those with MDD [35].

Our study took a different approach compared to previous investigations conducted in both Thai and other languages. In our study, we analyzed syntax based on participants’ responses to specific stimuli during the tests. This approach contrasts with previous research where syntactic analysis was conducted using notes, diaries, and Twitter datasets, which provided more freedom for individuals to express themselves through language [14,22,36].

Additionally, it is important to note that the Thai language has unique syntactic patterns. While some Thai sentences follow the S + V + O structure, Thai allows for the omission of pronouns. Thai speakers occasionally omit the topic when there is only one subject throughout the entire text or when the context is readily apparent. Therefore, merely counting and comparing the number of pronouns may not effectively differentiate between Thai depressed and non-depressed individuals. This phenomenon may be attributed to the relative simplicity of Thai syntax compared to English [37]. As a result, we had to employ additional criteria for assessing syntactic errors, such as word order and verb proficiency, to better determine syntax scores.

We also found a negative correlation between the severity of depression and syntax scores, suggesting that as the level of depressive symptoms increases, syntax tends to deteriorate. Consistent with the *t*-test outcomes between the depressed and non-depressed groups, the Early Reasoning test revealed the strongest association with the depressive scores. Notably, all subtests of the Early Reasoning and Comprehension tests displayed negatively significant correlations with depressive scores, albeit with small effect sizes. This observation might be attributed to the potential advantages of continuous variables, which often offer greater variability and a wider range of values. These attributes can enhance the likelihood of identifying statistically significant correlations when a genuine relationship exists between the variables. Interestingly, self-reported depression symptoms seemed to have a stronger statistical association with syntax performance compared to depression diagnosed by clinicians. This discrepancy was supported by a previous study [38].

Regarding reaction time, participants with depression generally displayed longer reaction times compared to those without depression. This could be attributed to impaired executive processes in major depressive disorder and difficulties in maintaining stable mental representations of incentives [39]. However, it is important to recognize that the type of test used may contribute to differences in response time. Most of the tests did not reach statistical significance in this regard, except for one—the puzzle test.

In terms of cognitive ability, the depressed group scored lower than the non-depressed group, confirming a difference in cognitive ability. It is worth noting that both groups did not differ in cognitive function scores assessed by the Mini-Cog, and neither group met the criteria for cognitive impairment. Therefore, this comparison of syntax was made between the two groups without the influence of cognitive impairment. The lower cognitive ability associated with depression is consistent with several related studies [40,41]. In our current study, depressed participants scored lower on all tests (except for Early Reasoning) compared to their non-depressed counterparts. These results are consistent with the findings obtained in other related research [42], which have established positive associations between specific brain regions (e.g., gyrus rectus, anterior cingulate, orbitofrontal volumes) and sequencing and nonverbal abstract reasoning scores, as well as negative associations with fluency error scores [43]. The lack of differentiation between the two groups in the Early Reasoning test may be attributed to cultural factors.

It is important to acknowledge that using psychological tests in research has its advantages and disadvantages. One drawback is that it constrains participants’ responses, limiting their ability to express themselves freely. This limitation can restrict researchers from conducting a more comprehensive analysis of the structure and arrangement of words and phrases within sentences, including aspects such as subject–verb agreement, word order, and grammatical rules governing sentence construction. However, utilizing these tests enables researchers to identify which assessments are effective in capturing syntactic issues. For example, the ‘laundry’ subtest within the Early Reasoning test can offer valuable insights into the differences in syntax usage between the two groups. Similarly, reaction time from the ‘puzzle’ subtest is found to be related to depression, whereas other tests were not. On the other hand, while the Early Reasoning test aimed to assess syntactic ability, the remaining components of the composite test—specifically Similarity, Vocabulary, Comprehension, and the Verbal Comprehension Index—could serve to distinguish the cognitive ability between depression and non-depression.

### 4.1. Research Implication

Despite the limitation of the sample size, this research contributes valuable insights into understanding elderly individuals who grapple with depression in the context of their syntactic language. This study furnishes evidence of language and late-life depression, revealing that individuals with depression exhibit more syntactic errors than their non-depressed counterparts in the Thai language. Furthermore, our findings suggest that certain tests may possess higher sensitivity to detect a syntactic difference between depression and non-depression by using a small sample, whereas some tests, such as Comprehension, may require a larger sample size. In addition, Similarity, Vocabulary, Comprehension, and the Verbal Comprehension Index can provide a cognitive ability score to distinguish between depression and non-depression. Consequently, these tests merit further research and refinement as potential screening tools for detecting depression.

Additionally, the authors recommend the development of Linguistic Inquiry and Word Count (LIWC) tools tailored to the Thai language or the creation of word analysis software and word count software designed specifically for the systematic analysis of Thai syntactic errors in late-life depression.

Presently, there is no available software for practitioners to perform these specific linguistic analyses effortlessly. However, the development of such a program falls within the purview of contemporary computational linguistics. If the outcomes of this study are validated through further research, the creation of a user-friendly, Windows-based syntax coding system could offer a valuable tool for language analysis in the realm of depression screening among older individuals.

### 4.2. Limitations

Despite conducting the research on-site at Maharaj Nakorn Chiang Mai Hospital, there were significant limitations in this study. Due to the small sample size, statistical significance cannot be securely confirmed in some tests. While the three-point syntax scoring system (2-1-0) offers simplicity in usability, it comes at the cost of reduced discriminatory power and sensitivity, particularly when applied to small sample sizes.

This study did not consider the medications received by the case group or potential comorbidities that might have influenced the outcomes. Additionally, although no difference in age and level of education was observed between the two groups, the effect of age range and education on the outcome variables was not elucidated.

Lastly, certain tests, like playing jigsaw puzzles, might be influenced by cultural factors as they seem unfamiliar to many older Thai participants. Other cultural-related factors were not documented or recorded.

## 5. Conclusions

This study has uncovered a notable presence of syntax problems in patients diagnosed with major depressive disorder. Older individuals with depression exhibited a tendency to employ shortened sentences by omitting certain structural elements. Additionally, despite the absence of statistical significance, they tended to demonstrate longer reaction times. Specific tests could identify syntactic differences, while most composite tests effectively distinguished cognitive abilities between the two groups. However, due to the limited sample size, some tests were unable to detect differences. Further research should aim to develop a practical, user-friendly screening tool for identifying depression and integrating syntactic analysis with cognitive ability cut-off scores.

## Figures and Tables

**Table 1:** Socio-demographic data.

Variables	Depressed	Non-Depressed	*t*-Test
	Mean ± SD or *n* (%)	Mean ± SD or *n* (%)	
Sex			Chi-square (1) ^a^ = 0.00, *p* = 1.000
Male	9 (40.9%)	9 (40.9%)	
Female	13 (59.1%)	13 (59.1%)	
Age	67.22 ± 6.32	67.86 ± 5.21	*t* (42) ^a^ = −0.364 *p* = 0.717
Education			Fisher’s exact test = 6.310, *p* = 0.186
Primary	7 (31.8%)	1 (4.5%)	
Secondary	4 (18.2%)	6 (27.3%)	
High school	3 (18.2%)	3 (13.6%)	
Bachelor’s degree	4 (13.6%)	5 (22.7%)	
Above bachelor’s degree	4 (18.2%)	7 (31.8%)	
Dialect			Chi-square (3) ^a^ = 1.059, *p* = 0.787
Northern	13 (50%)	13 (50%)	
Southern	9 (50%)	9 (50%)	
Income			Chi-square (4) ^a^ = 4.947, *p* = 0.293
USD 0–137.67	11 (50%)	4 (18.2%)	
USD 137.7–275.35	2 (9.1%)	4 (18.2%)	
USD 275.38–413.02	2 (9.1%)	4 (18.2%)	
USD 413.05–550.7	1 (4.5%)	2 (9.1%)	
USD 550.7+	6 (27.3%)	8 (36.4%)	
Living status			Chi-square (1) ^a^ = 1.100 *p* = 0.294
Living with other	20 (90.9%)	19 (86.4%)	
Living alone	2 (9.1%)	3 (13.6%)	
Alcohol use in the past 12 months			Chi-square (1) ^a^ = 2.071, *p* = 0.150
Yes	3 (30%)	7 (70%)	
No	19 (55.9%)	15 (44.1%)	
Marital status			Chi-square (2) ^a^ = 4.401, *p* = 0.111
Married	16 (72.7%)	15 (68.2%)	
Divorced	6 (27.3%)	3 (13.6%)	
Other relationship	0 (0.0%)	4 (18.2%)	

^a^ Degree of freedom, 1 US Dollar = 35.2 Thai Baht on the exchange rate at December 2023.

**Table 2:** Syntax scores between depressed and non-depressed groups.

Tests	Depressed (Mean ± SD)	Non-Depressed (Mean ± SD)	Mean Difference	Bootstrap 95% Confidence Interval		Cohen’s d
				Lower	Upper	
Similarity	1.57 ± 0.31	1.60 ± 0.23	0.02389	−0.14767	0.20327	0.079
Vocabulary	1.59 ± 0.29	1.64 ± 0.32	−0.04917	−0.21768	0.13059	0.162
Comprehension	1.81± 0.23	1.89 ± 0.15	−0.07762	−0.19053	0.03193	0.395
Total scores of Early Reasoning	1.53 ± 0.40	1.76 ± 0.28	−0.22535	−0.43717	−0.02739	0.646
- Cat and Ball	1.41 ± 0.50	1.63 ± 0.49	−0.222	−0.505	0.077	0.447
- Laundry	1.45 ± 0.51	1.81 ± 0.39	−0.357	−0.636	−0.106	0.785
- Puzzle	1.73 ± 0.46	1.91 ± 0.29	−0.182	−0.408	0.036	0.474

**Table 3:** Correlation between the TGDS and syntax scores of each subtest.

	*r*	Bootstrap 95% Confidence Intervals	
Similarity	−0.048	−0.394	0.267
Vocabulary	−0.208	−0.520	0.082
Comprehension	−0.289	−0.509	−0.011
Early Reasoning	−0.426	−0.647	−0.147
- Cat and Ball	−0.307	−0.571	−0.018
- Laundry	−0.453	−0.681	−0.184
- Puzzle	−0.331	−0.584	−0.002

**Table 4:** Reaction time between depressed group and non-depressed group.

Variables	Depressed	Non-Depressed	Mean Difference	Bootstrap 95% Confidence Intervals		Cohen’s d
				Lower	Upper	
Similarity	5.23 ± 3.51	5.24 ± 4.27	0.16	−2.08	2.32	0.04
Vocabulary	3.98 ± 2.55	2.97 ± 1.9	0.93	−0.37	2.26	0.43
Comprehension	5.43 ± 3.20	4.28 ± 2.98	1.36	−0.46	3.20	0.45
- Cat	5.86 ± 4.71	6.21 ± 10.13	−0.31	−5.59	3.57	−0.04
- Laundry	9.90 ± 7.24	6.59 ± 6.10	3.61	−0.57	7.78	0.54
- Puzzle	6.31 ± 3.3	4.31 ± 2.78	2.07	0.17	4.01	0.67

**Table 5:** The cognitive ability scores between depressed group and non-depressed group.

Variables	Depressed	Non-Depressed	Mean Difference	Bootstrap 95% Confidence Intervals		Cohen’s d
				Lower	Upper	
Similarity	4.91 ± 2.96	7.23 ± 2.58	−2.13	−3.79	−0.36	0.73
Vocabulary	6.77 ± 2.89	8.91 ± 2.41	−2.22	−3.75	−0.55	0.85
Comprehension	6.82 ± 3.06	9.50 ± 3.08	−2.68	−4.45	−0.88	0.87
Early Reasoning	4.09 ± 1.74	4.27 ± 1.32	−0.18	−1.09	0.71	0.12
Verbal Comprehension Index			Chi-square (4) = 8.650, *p* = 0.07			
Above average	1 (50%)	1 (50%)				
Average	6 (31.6%)	13 (68.4%)				
Borderline	6 (75%)	2 (25%)				
Lower than average	3 (37.5%)	5 (62.5%)				
Extremely low	6 (85.7%)	1 (14.3%)				

## Data Availability

The datasets used and/or analyzed during the current study are available from the corresponding author upon reasonable request.
